# Monitoring fine root growth to identify optimal fertilization timing in a forest plantation: A case study in Northeast Vietnam

**DOI:** 10.1371/journal.pone.0225567

**Published:** 2019-11-25

**Authors:** Tran Van Do, Nguyen Toan Thang, Vu Tien Lam, Dang Van Thuyet, Phung Dinh Trung, Tran Hoang Quy, Nguyen Thi Thu Phuong, Ly Thi Thanh Huyen, Nguyen Huu Thinh, Nguyen Van Tuan, Dao Trung Duc, Dang Thi Hai Ha, Duong Quang Trung, Ho Trung Luong, Nguyen Thi Hoai Anh, Patrick Nykiel

**Affiliations:** 1 Silviculture Research Institute, Vietnamese Academy of Forest Sciences, Hanoi, Vietnam; 2 Independent Australian Researcher, Vietnamese Academy of Forest Sciences, Hanoi, Vietnam; University of Vigo, SPAIN

## Abstract

Fertilizer is applied widely to improve the productivity of plantations. Traditionally, fertilization is conducted in spring and/or in the early rainy season, and it is believed to support the growth of planted trees in the growing season. Little attention to date has been paid on identification of the optimal timing of fertilization and fertilizer dose. In this study, application of the fine root monitoring technique in identifying optimal fertilization timing for an *Acacia* plantation in Vietnam is described. The study used two fertilizer doses (100 and 200 g NPK/tree) and three fertilization timings (in spring; in the early rainy season; and based on the fine root monitoring technique to identify when the fine roots reach their growth peak). As expected fertilization timings significantly affected growth and above-ground biomass (AGB) of the plantation. Fertilization based on the fine root monitoring technique resulted in the highest growths and AGB, followed by fertilization in the early rainy season and then in spring. Applying fertilizer at 200 g NPK/tree based on the fine root monitoring technique increased diameter at breast height (DBH) by 16%, stem height by 8%, crown diameter (D_c_) by 16%, and AGB by 40% as compared to early rainy season fertilization. Increases of 32% DBH, 23% stem height, 44% D_c_, and 87% AGB were found in fertilization based on fine root monitoring technique compared to spring fertilization. This study concluded that forest growers should use the fine root monitoring technique to identify optimal fertilization timing for higher productivity.

## Introduction

Total area of industrial plantation forest in the world is 54.3 million ha [1]. Asia has the highest plantation area, at 17.7 million ha. By 2015, planted forest areas accounted for 7 percent of the world’s forest areas [2]. It is estimated that the global plantation area may reach 90 million ha by 2050 [1]. Increasing productivity [3] and practicing sustainable management are necessary to remain plantation areas, reduce deforestation [4], and overcome timber shortfalls [5]. In plantation, nutrients are lost due to soil erosion and biomass remove in logging, leading to decreased productivity of the following rotations [6]. Fertilization has been applied in many species and regions globally to increase plantation productivity [7; 8], and it is a viable silvicultural option in plantation management and development [9]. Nutrient availability is often a significant limiting factor in plantation growth [10], and nutrients usually become deficient when the trees are developing their canopy. Macronutrients [11; 12] such as nitrogen (N), phosphorus (P), and potassium (K) are the key limiting nutrients in many sites because they are often taken up in large quantities [13].

Fertilizers could be applied annually during and/or after planting to support tree growth [14–16]. The main reason for fertilization of any forest plantation is to provide planted trees with nutrients for improving growth [17]. Therefore, growers expect planted trees to be able to absorb as much applied fertilizer as possible [18]. Estimating how much applied fertilizer planted trees can absorb is not easy work. However, if the same fertilizer type and dose were applied in the same plantation at different times t_1_ and t_2_, and time t_1_ of the application resulted in significantly better growth (e.g., stem height, diameter at breast height, crown diameter, above-ground biomass), it indicated that the planted trees in time t_1_ fertilization absorbed applied fertilizer more efficiently. Traditionally, fertilizer is applied either in spring or early in the rainy season in areas where there are four seasons a year, and early in the rainy season in areas where there are two distinct seasons; one dry and one rainy [19; 20], as such fertilizer could support planted trees growing better in the growing season. Study with *Pinus taeda* L. plantation indicated that fertilizers could be applied at planting or early post-planting and at canopy closure to enhance straw production and maintain stand vigor [21; 22]. While time of year is not critical with P fertilization in *P*. *taeda* plantation [21]. In addition, fertilization after thinning leads to exceeded stand N demand and therefore high nutrient loss in *P*. *taeda* plantation [23]. Different fertilization timings were also tested at *Eucalyptus urophylla* plantations in southern China, indicating significant effects on growths and above-ground biomass [24]. Fine roots are roots of ≤2 mm in diameter that absorb water and nutrients to support the growth of trees [25; 26]. The lifespan of fine roots is quite short compared to the life of the tree, as these fine roots live only for weeks to several months [27; 28]. Therefore, the fine root growth of planted trees could be considered as an indicator for the timing of fertilization. In winter, the growth of trees slows down, and there is less development of fine roots in temperature *Quercus serrata* plantation [27] and in a secondary forest of *Q*. *serrata* [29]. Accordingly, it is pointless to apply fertilizer in such areas in winter to plantations. In late spring and the early summer/rainy season, tree growth accelerates, and new leaves and fine roots appear [27]. Application of fertilizer at this time is more effective, because the increased number of fine roots facilitates more efficient uptake of fertilizer.

The objectives of the present study were: (1) to describe the fine root monitoring technique for identifying optimal timing of fertilization; and (2) to test the effects of different fertilization timings on tree growth in an *Acacia mangium* Willd. plantation in northeast Vietnam.

## Materials and methods

### Description of study site

**Fig 1:**
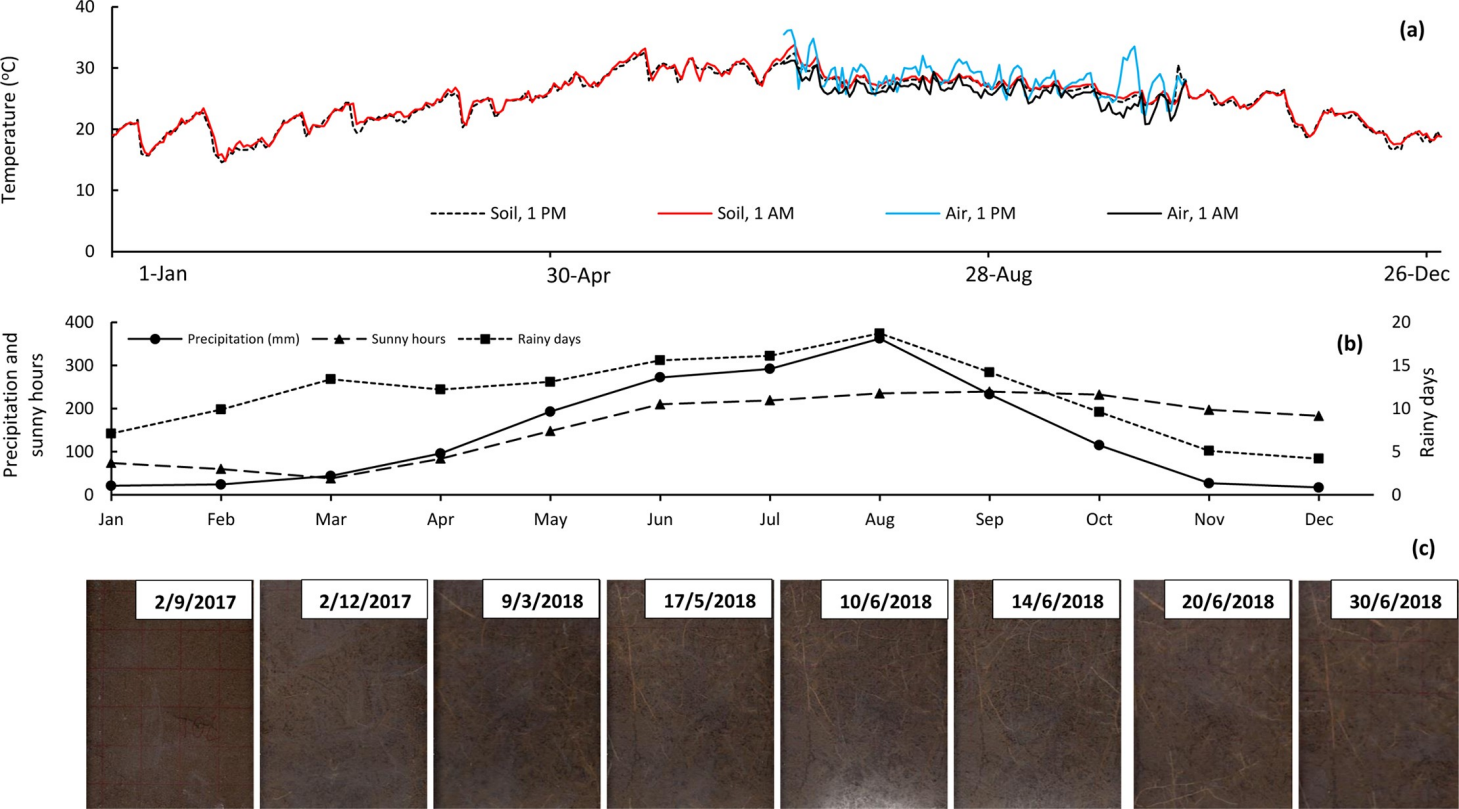
Changes of daily temperature in 2018 **(a)**, average of 10-year records of precipitation, sunny hours and rainy days **(b)**, and scanned images of fine root growth **(c).** Early rainy season starts late May/early June when precipitation is higher than 100 mm/month. While a precipitation > 20 mm/day is known as heavy rain.

This study was conducted at the Forest Experiment Station (FES), the College of Agriculture and Forestry Northeast in Uong Bi City, Quang Ninh Province Vietnam. Acacia plantations have been established widely in the vicinity of the FES by private companies and local farmers. Uong Bi City has monsoon climate conditions, with an annual temperature of 22.2°C and air humidity of 81% [30]. There are four distinct seasons: spring (Mar–May); summer (Jun–Sep); autumn (Sep–Nov); and winter (Dec–Jan). There are six to seven hours of sunshine per day in summer and three to four hours per day in winter, with an average of 24 sunny days per month. The rainy season is between June and August. The amount of total annual precipitation is 1,600–2,200 mm, and there are on average 153 rainy days per year (Fig 1). There are four months (Dec–Feb) when precipitation is <40 mm, with fewer than 10 rainy days per month, and there are six months (May–Oct) when precipitation is >100 mm, with more than 10 rainy days per month. There are four months (Jan–Apr) that have <85 hours of sunshine per month and eight months (May–Dec) that have >100 hours of sunshine per month. Details of air and soil temperatures in 2018 are shown in Fig 1A; which were recorded by automatic air and soil temperature recorders (Thermo Recorder T&D, TR-51*i*) set at 0.5 m above the soil surface and at 15 cm below the soil surface.

The site for the experiment was located on the flat land with a slope of < 2°. The site was classified as bare land with some *Acacia mangium* and *Eucalyptus urophylla* trees and other shrubs that were lower than 3 m tall. The site was cleared and burned to prepare it for the experiment. The soil was classified as Ferralic Acrisol [31] with a depth of 0.7 m. Soil sample analysis indicated a pH of 3.5–3.6, organic matter content of 3.5%–3.8%, N content of 0.09%–0.12%, P content of 0.23–0.24 mg P_2_O_5_/100 g, K content of 4.19–4.21 mg K_2_O/100 g, coarse sand (2–0.2 mm) of 41.5%–42.0%, fine sand (0.2–0.02 mm) of 30.1%–30.5%, loam (0.02–0.002 mm) of 7.8%–7.9%, and silt (<0.002 mm) of 3.5%–3.7%.

### Experiment design

*Acacia mangium* was used, because this species has been widely planted for pulp and timber both in the northeast and across Vietnam. Four-month-old seedlings with a stump diameter/D_o_ (diameter measured at collar root) of 3.7–3.8 mm and a height of 0.57–0.59 m were used. The seedlings were produced from quality-controlled seeds imported from Australia. Planting pits with sizes of 40 × 40 × 40 cm were prepared. Trees were planted on September 2, 2017, near the end of the rainy season.

**Fig 2:**
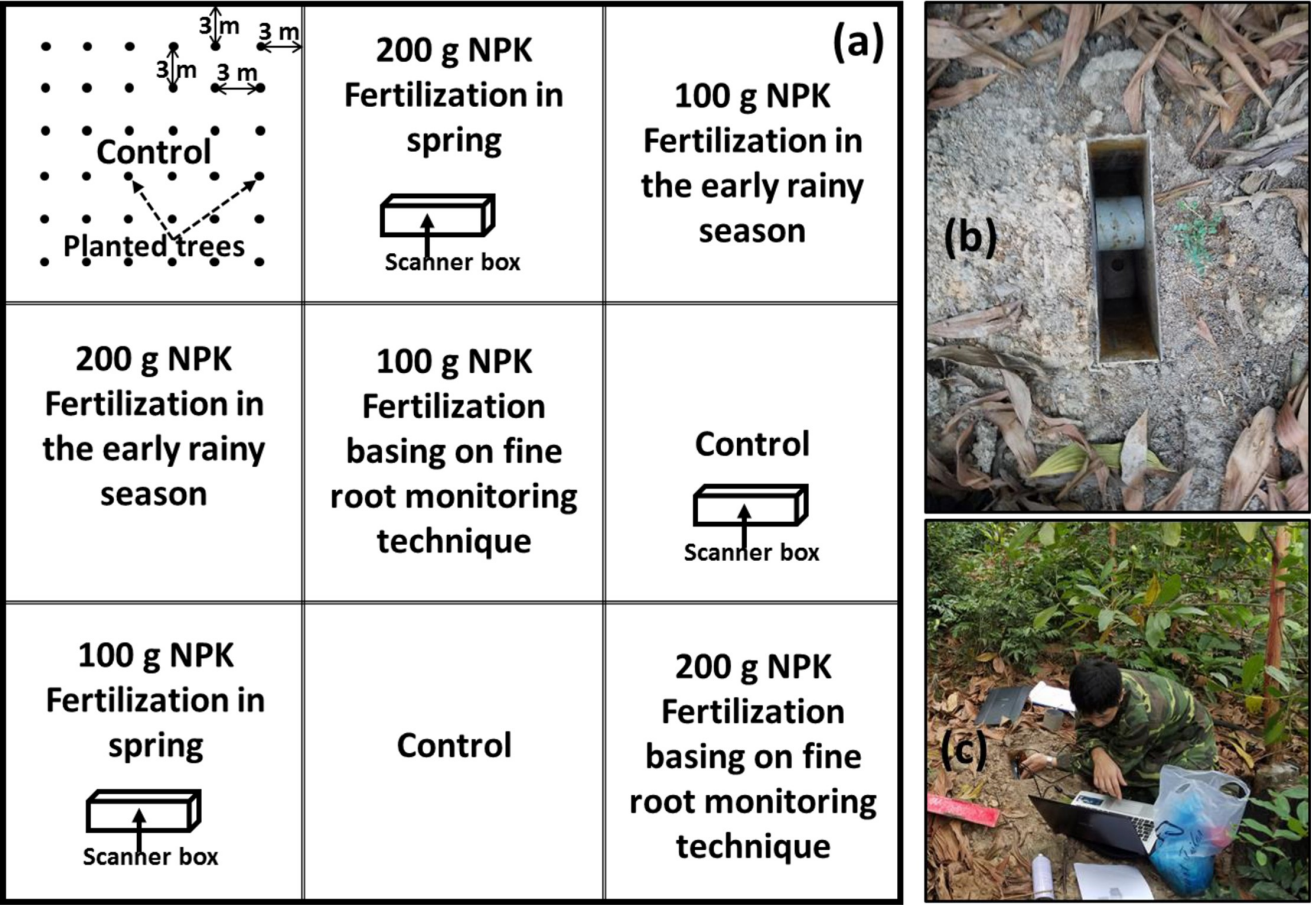
Experiment layout **(a)**, a buried transparent scanner box **(b)**, and scanning **(c)**.

An experiment to identify optimal fertilization timings is valuable only when fertilization positively impacts the growth of planted trees [32]. Therefore, experiments examining both the dose of fertilizer and the timing of the fertilization were conducted. There were three treatments of fertilizer dose: (1) application of 100 g NPK/tree (NPK with a ratio of 16:16:8); (2) application of 200 g NPK/tree; and (3) a control (no application of fertilizer). There were three treatments of fertilization timing: (a) in spring; (b) in the early rainy season; and (c) at a time based on observation of fine root growth by a fine root monitoring technique. There were 9 subplots in a main plot (Fig 2. The individual in this manuscript has given written informed consent (as outlined in PLOS consent form) to publish these case details), each subplot contained 36 trees (6 × 6) planted in 3 × 3 m spacing. The space between subplots was 6 m, which ensured no cross-interference between subplots. There were three main plots as three replicates and the experiment was designed in a block, containing 27 subplots.

The dose of fertilizer applied was divided equally over four pits at the north, south, east, and west of a stump of *A*. *mangium* tree. The distance from stump to pits was determined by observing the extent of fine root growth. Pits of 10–15 cm depth were made, and fertilizer was inserted and then covered with fine soil. Vegetation near the pits was cleared. All fertilizer was applied only one time in each treatment.

### Identifying fertilization timing by the fine root monitoring technique

Techniques for observing fine root growth were developed by Ferguson et al. [33], Dannoura et al. [34], Di Iorio et al. [35], Cislaghi et al. [36], and Vergani et al. [37]. Ferguson et al. [33] used the rhizotron technique: burying a transparent tube in the soil and taking photographs from the tube periodically to observe fine root growth. Dannoura et al. [34] buried a scanner, which was protected in a transparent plastic box below the soil surface, and fine root images were periodically scanned by connecting the scanner to an Laptop. Through a series of scanned images, the growth of already formed and new fine roots can be observed and measured nondestructively (Fig 1C).

In this study, scanner boxes made of transparent plastic were buried in three subplots (Fig 2A and 2B) in September 2017. The box has dimensions of 25 × 37 × 7 cm. It was buried vertically to the soil with a depth of 37 cm. While an A-4 scanner (CanoScan LiDE 210) has dimensions of 25 × 36.5 × 3.5 cm, which can fit well inside the box. In each subplot, three boxes were buried at 0.5 m, 1.0 m, and 1.5 m distance from the stump of the *A*. *mangium* trees. The boxes had lids and were covered with fine soil to maintain darkness inside them. A mobile A4-scanner was placed in the box and connected to a Laptop through a USB cable for scanning (Fig 2C).

In this study, roots with diameter ≤2 mm are considered as fine roots [25; 26]. Fine root length can be measured nondestructively for time-intervals through series of images captured periodically (Fig 1C) by using Rootfly (SmartRoot), a semi-automated image analysis software [38]. The images of fine root growth were scanned on September 2, 2017; December 2, 2017; March 9, 2018; April 10, 2018; May 2, 2018; May 17, 2018; June 1, 2018; June 10, 2018, June 14, 2018; June 20, 2018; and June 30, 2018. The highest value of fine root length was determined as a peak of fine root growth and it was a fertilization timing by the fine root monitoring technique.

### Data collection and above-ground biomass estimation

Stump diameter (D_o_, measured at collar root) of *A*. *mangium* trees was measured at 3 and 6 months after planting (trees were planted in September 2017); diameter at breast height (DBH) was measured at 9, 12, and 15 months after planting; stem height was measured at 3, 6, 9, 12, and 15 months after planting; and crown diameter was measured at 15 months after planting. The above-ground biomass (AGB) of each tree stem was estimated based on DBH measured at 15 months by applying allometry in Eq 1 [39]. Then, AGB of each replicate was total biomass of all stems in that replicate (subplot; Fig 2).

$$AGB=0.223*DBH^{2.251}.$$ (1)

The allometry (Eq 1) was established based on destructive sampling 15 *Acacia mangium* trees aging 3, 4 and 5 years old. The sampling trees had DBH ranging from 4.1 cm to 12.7 cm. The relationship has regression/R^2^ of 0.97 and *p* <0.001.

### Statistical analysis

Growth parameters (D_o_, DBH, stem height, and crown diameter) are reported as the mean of all measured individuals (n = 108) with its standard error (SE). AGB is reported as the mean of three replicates with SE. While comparison of growths and AGB between fertilization timings (fine root monitoring technique and early rainy season; fine root monitoring technique and spring) is reported as percentage.

A two-way Analysis of Variance (ANOVA) [40] was performed to identify the differences between the factors (fertilizer dose and fertilization timing). To obtain maximum power and robustness of the test, equal sample sizes were used. In the case of overall significant differences in the group means, Duncan’s multiple-range post hoc test was performed to determine the differences between means for each growth parameter in each measured time [41]. All analyses were conducted using SAS 9.2 (SAS Institute Inc., Cary, NC, USA).

## Results

### Fertilization timings

**Fig 3:**
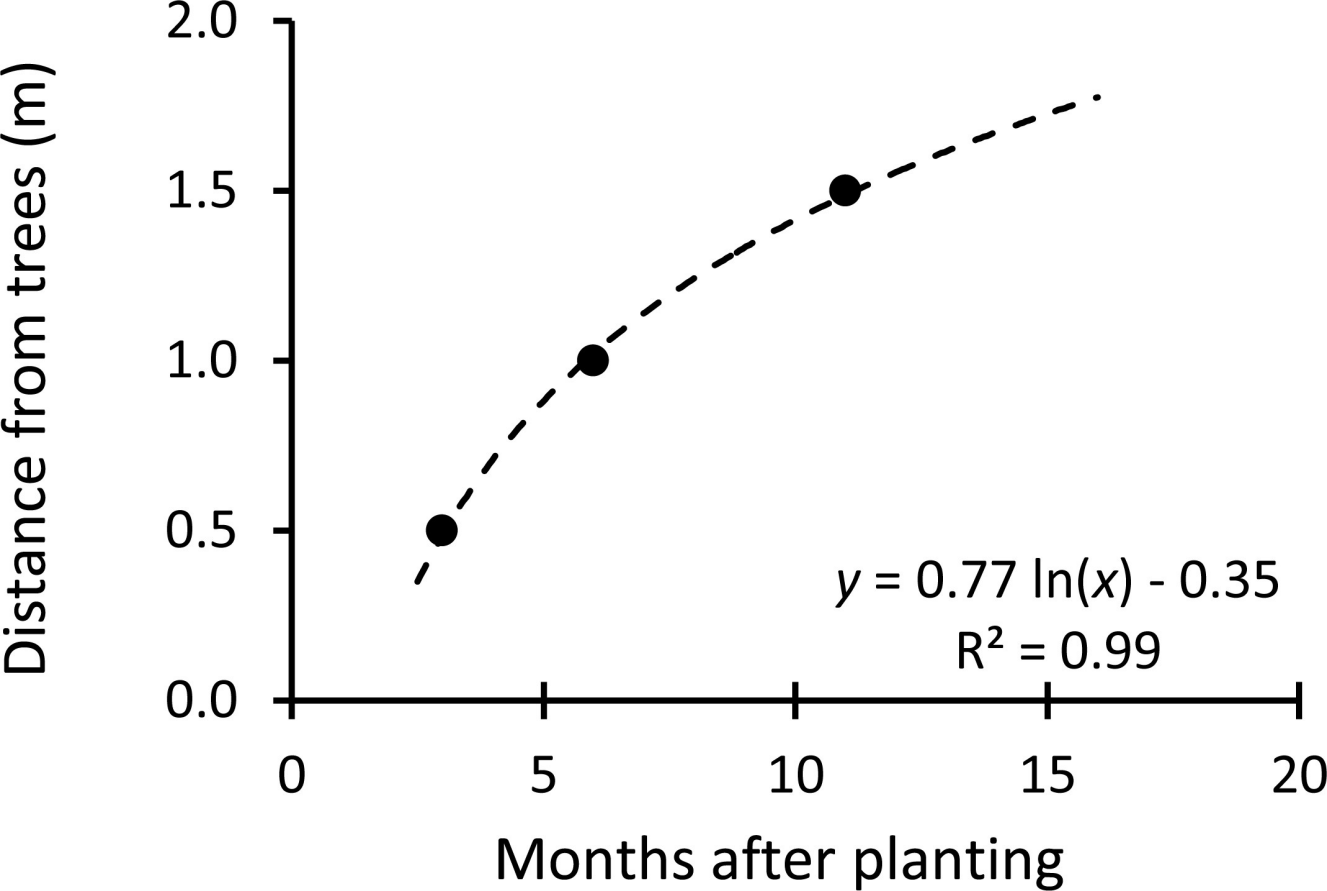
Fine root growth by a timeline.

Fine roots grew further as trees were getting older (Fig 3). Fine roots grew to a distance of 0.5 m from stumps at 3 months after planting, to 1 m at 6 months after planting, and to 1.5 m at 9 months after planting. In spring fertilization, therefore, fertilizer was applied on March 3, 2018 at a distance of 1.0 m from the trees. In early rainy season fertilization, fertilizer was applied on June 2, 2018 at a distance of 1.2 m from the trees.

Observation of the scanned images (Fig 1C) indicated that the least fine root growth occurred between December 2, 2017 and March 3, 2018. The growth increased between March 3 and May 17, 2018, and between May 17 and June 10, 2018. It then achieved maximum between June 10 and June 14, 2018. Therefore, fertilization timing based on the fine root monitoring technique was decided on June 15, 2018. Fertilizer was applied at a distance of 1.3 m from the trees (Fig 3).

### Tree growth from different doses of fertilizer

At planting, there were no significant differences (*p* >0.43) of D_o_ and height, and this confirmed that the initial sizes of seedlings had no effect on the results of the experiment (Table 1). At 3, 6, 9, 12, and 15 months after planting, fertilization resulted in significantly greater growths (*p* <0.04) than that in control. At 15 months after planting, non-fertilized trees achieved averages of 39.0 ±0.54 mm DBH, 3.02 ±0.03 m height, and 1.70 ±0.02 m D_c_. Meanwhile, the trees in the 200 g NPK fertilization reached averages of 67.38 ±0.33 mm DBH, 4.40 ±0.02 m height and 2.50 ±0.01 m D_c_, and the trees in the 100 g NPK fertilization reached averages of 61.56 ±0.54 mm DBH, 3.85 ±0.02 m height and 2.00 ±0.01 m D_c_, which were fertilized by fine root monitoring technique.

**Table 1:** Growths at 3-month intervals for different fertilizer doses and fertilization timings.

	D_o_ (mm)			DBH (mm)			H (m)						D_c_ (m)
Months after planting Fertilization timing	0	3	6	9	12	15	0	3	6	9	12	15	15
Fertilizing 100 g NPK/tree												
Monitoring technique (6/15/2018)	3.82 ±0.05	16.81^a^ ±0.12	24.63^a^ ±0.15	16.44^a^ ±0.19	53.30^a^ ±0.47	61.56^a^ ±0.54	0.58 ±0.01	1.23^a^ ±0.01	1.52^a^ ±0.01	2.38^a^ ±0.01	3.61^a^ ±0.04	3.85^a^ ±0.02	2.00^a^ ±0.01
Early rainy season (6/2/2018)	3.75 ±0.04	16.65^a^ ±0.11	24.20^a^ ±0.14	16.32^a^ ±0.18	48.73^b^ ±0.33	57.98^b^ ±0.37	0.57 ±0.00	1.15^a^ ±0.01	1.47^a^ ±0.01	2.41^a^ ±0.01	3.47^b^ ±0.02	3.75^b^ ±0.03	1.88^b^ ±0.01
Spring (3/9/2018)	3.8 ±0.04	16.72^a^ ±0.10	24.53^a^ ±0.15	21.33^b^ ±0.18	48.12^b^ ±0.33	56.58^b^ ±0.37	0.57 ±0.00	1.18^a^ ±0.01	1.49^a^ ±0.02	2.49^b^ ±0.02	3.39^c^ ±0.03	3.65^c^ ±0.02	1.71^c^ ±0.01
	Fertilizing 200 g NPK/tree												
Monitoring technique (6/15/2018)	3.84 ±0.02	16.68^a^ ±0.11	23.91^a^ ±0.17	16.23^a^ ±0.18	59.55^c^ ±0.40	67.38^c^ ±0.33	0.59 ±0.00	1.16^a^ ±0.01	1.48^a^ ±0.01	2.39^a^ ±0.02	4.02^d^ ±0.01	4.40^d^ ±0.02	2.50^d^ ±0.01
Early rainy season (6/2/2018)	3.88 ±0.02	16.71^a^ ±0.12	24.06^a^ ±0.18	16.23^a^ ±0.18	47.53^b^ ±0.47	57.97^b^ ±0.53	0.57 ±0.01	1.06^a^ ±0.01	1.40^a^ ±0.01	2.36^a^ ±0.02	3.76^e^ ±0.02	4.09^e^ ±0.03	2.15^a^ ±0.01
Spring (3/9/2018)	3.79 ±0.03	16.54^a^ ±0.11	23.81^a^ ±0.17	22.16^b^ ±0.17	44.34^d^ ±0.79	50.98^d^ ±0.44	0.57 ±0.00	1.10^a^ ±0.01	1.39^a^ ±0.01	2.56^b^ ±0.02	3.24^f^ ±0.01	3.57^c^ ±0.02	1.74^c^ ±0.01
	Control (no fertilization)												
	3.78 ±0.03	10.91^b^ ±0.13	16.19^b^ ±0.21	9.38^d^ ±0.20	31.04^e^ ±0.43	39.00^e^ ±0.54	0.57 ±0.00	0.81^b^ ±0.01	1.03^b^ ±0.01	1.83^c^ ±0.02	2.82^g^ ±0.02	3.02^f^ ±0.03	1.70^c^ ±0.02

D_o_ is stump diameter; DBH is diameter at breast height; H is stem height; and D_c_ is crown diameter. A two-way ANOVA (fertilizer dose and fertilization timing) analysis indicates *df*_within_ = 642, *df*_total_ = 647, and *p* <0.05. Different letters ^a, b, c, d, e, f, g^ in a column indicate significant difference of means by Duncan’s multiple-range test.

### Tree growth from different fertilization timings

**Fig 4:**
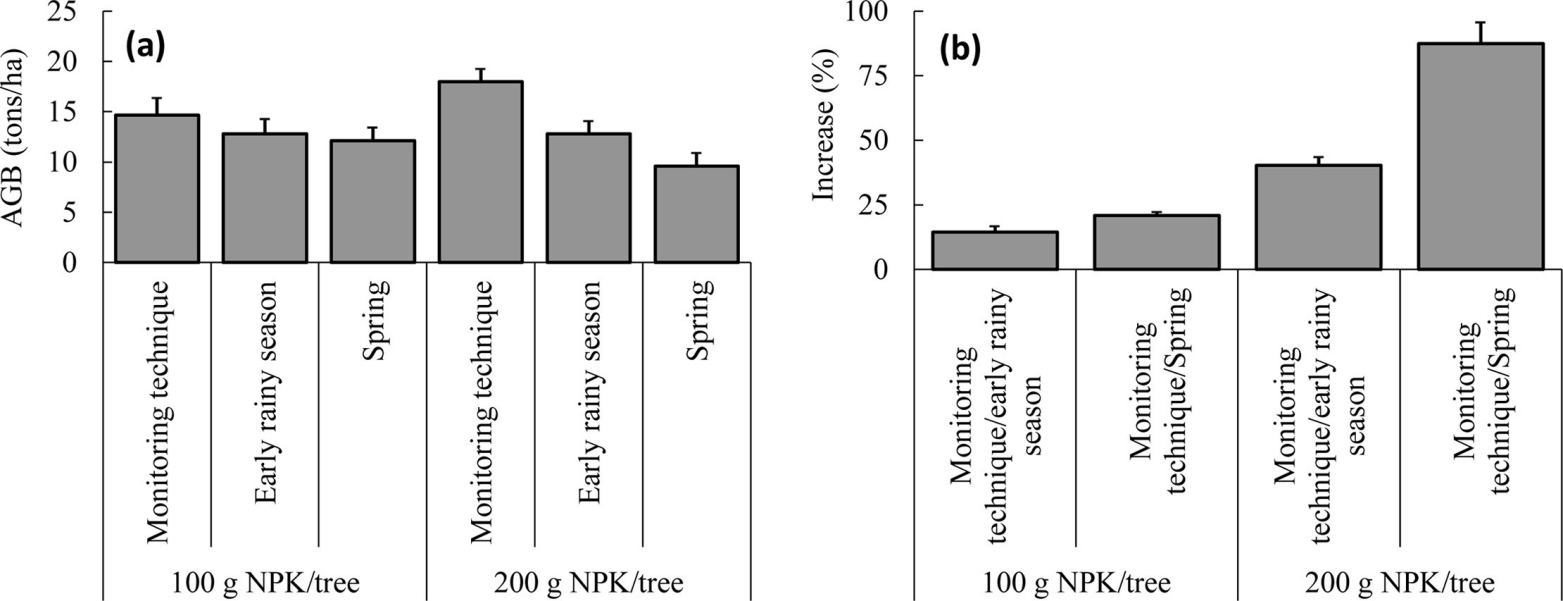
Above-ground biomass/AGB of *A*. *mangium* plantation at 15 months after planting **(a)** and comparison (%) of AGB between fertilization timings; the fine root monitoring technique and the early rainy season (monitoring technique/early rainy season) and between the fine root monitoring technique and spring (monitoring technique/Spring) **(b)**.

In the application of 100 g NPK/tree (Table 1), there were no significant differences (*p* >0.21) in growths among the three fertilization timings at 3 and 6 months after planting, since fertilizer had not yet been applied. Fertilizer was applied on March 9, 2018 in the spring fertilization. Nine months after planting, growths in the spring fertilization (21.33 ±0.18 mm DBH and 2.49 ±0.02 m height) were significantly higher (*p* <0.03) than growths in both the early rainy season fertilization (16.32 ±0.18 mm DBH and 2.41 ±0.01 m height) and fertilization based on the fine root monitoring technique (16.44 ±0.19 mm DBH and 2.38 ±0.01 m height). Fertilizer was applied on June 2, 2018 in the early rainy season fertilization and on June 15, 2018 in the fine root monitoring technique fertilization. At 12 months after planting, the DBH in the fine root monitoring technique fertilization (53.30 ±0.47 mm) was significantly higher (*p* <0.05) than DBH in the early rainy season (48.73 ±0.33 mm) and spring fertilizations (48.12 ±0.3 mm). The difference between the two later fertilizations was not significant (*p* >0.2). The difference in height among the three fertilization timings was significant (*p* <0.05), with the tallest trees belonging to the fine root monitoring technique fertilization (3.61 ±0.04 m), followed by the early rainy season (3.47 ±0.02 m) and spring fertilizations (3.39 ±0.03 m). A similar pattern of differences (*p* <0.05) in DBH and height among the three fertilization timings was observed at 15 months after planting. Additionally, the difference of D_c_ was significant (*p* <0.04), with the largest D_c_ in the fine root monitoring technique fertilization (2.00 ±0.01 m), followed by the early rainy season (1.88 ±0.01 m) and spring fertilizations (1.71 ±0.01 m). At 15 months after planting, there were significant differences (*p* <0.05) in AGBs among the three fertilization timings (Fig 4A), with the highest AGB in the fine root monitoring technique fertilization, followed by the early rainy season and spring fertilizations.

The patterns of change and differences in growths among the three fertilization timings with application of 200 g NPK/tree were similar to that with application of 100 g NPK/tree (Table 1). The growths (DBH, height, and D_c_) were highest in the spring fertilization at 9 months after planting and highest in the fine root monitoring technique fertilization at 12 and 15 months. The AGBs of the three fertilization timings were significantly different (*p* <0.05) at 15 months after planting (Fig 4A).

Regardless of the doses of fertilizer applied, fertilization timing based on the fine root monitoring technique increased growths by 2.7%–16.3% (Table 2) and AGB by 14.4%–40.3% (Fig 4B) at 15 months after planting compared to that in the early rainy season fertilization. The rises were even higher when comparing between the fine root monitoring technique and spring fertilizations. The figures ranged from 5.5%–43.7% for growths and 20.9%–87.4% for AGB at 15 months after planting.

**Table 2:** Increase (%; ±SE) of growths between fertilization timings of the fine root monitoring technique compared to that of the early rainy season and spring fertilizations with different fertilizer doses at 15 months after planting.

Comparison	DBH	Height	D_c_
	Fertilizing 100 g NPK/tree		
Monitoring technique/the early rainy season	6.2 ±0.58	2.7 ±0.19	6.4 ±0.57
Monitoring technique/spring	8.8 ±0.76	5.5 ±0.48	17.0 ±1.20
	Fertilizing 200 g NPK/tree		
Monitoring technique/the early rainy season	16.2 ±1.18	7.6 ±0.71	16.3 ±1.17
Monitoring technique/spring	32.2 ±2.11	23.2 ±1.98	43.7 ±2.78

**Fig 5:**
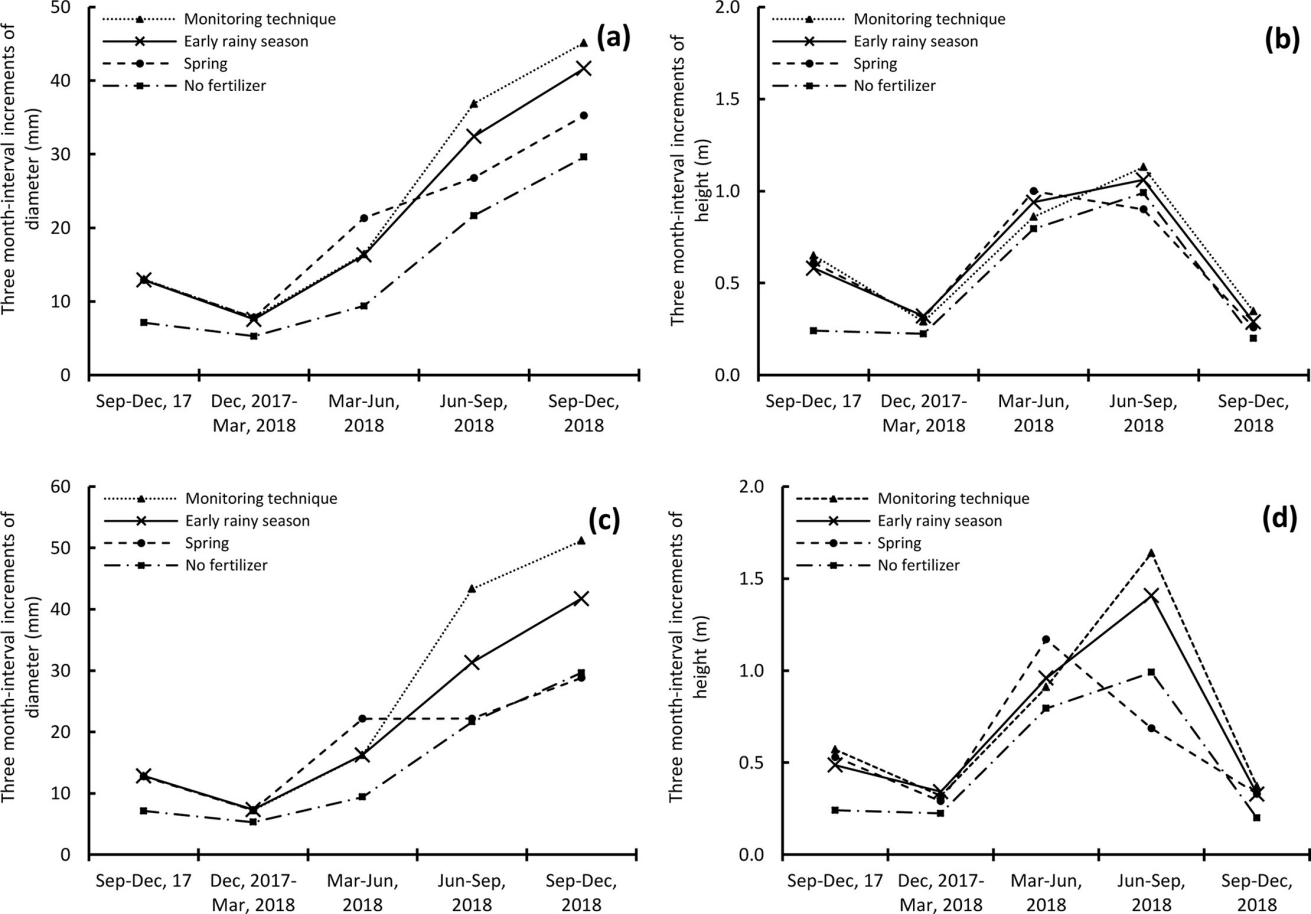
**Three-month interval increments of diameter at breast height (DBH) and stem height.** In **(a)** and **(b)** 100 g NPK/tree was applied. In **(c)** and **(d)** 200 g NPK/tree was applied.

Incremental values of DBH and height at three-month intervals are important to understand the direct impact of fertilization on tree growth. Regardless of fertilizer doses and fertilization timings, increases in DBH were lowest during December 2017–March 2018 (Fig 5A and 5C), followed by September–December 2017, March–June 2018, June–September 2018 and September–December 2018. In terms of height, increments were lowest during December 2017–March 2018 (Fig 5B and 5D), followed by September–December 2018, September–December 2017, March–June 2018, and June–September 2018. Irrespective of time intervals, control had increments lower than those of fertilizations for both DBH and height (Fig 5). In all the three fertilization timings, increments were highest during March–June 2018 for the spring fertilization. During June–September and September–December 2018, the highest increments for both DBH and height were observed in the fine root monitoring technique fertilization, followed by the early rainy season and spring fertilizations (Fig 5).

## Discussion

Fertilizer applied to a plantation is taken up by the planted trees and other vegetation [42] and is lost due to soil erosion through heavy rain, leaching, and/or emission [43]. Therefore, fertilization in heavy rains (a precipitation >20 mm/day is known as heavy rain; Fig 1) results in high loss of fertilizer. Meanwhile, fertilization at a time of maximum growth of other vegetation also leads to high loss of fertilizer. In this study, fertilization in spring seems to have avoided heavy rain (Fig 1B). However, the planted *A*. *mangium* trees were not ready to take up the applied fertilizer, both because their fine root numbers were limited (Fig 1C) and because spring is the time for maximum growth of grasses and other vegetation at the study site [31]. Therefore, in spring, there is a high rate of loss of applied fertilizer due to grass uptake. Conversely, fertilization in the early rainy season in this study led to higher fertilizer loss through soil erosion and leaching. However, due to the site being flat, the loss rate may be low. There were low fine roots on March 9 and June 2 (Fig 1C), leading to low fertilizer uptake. Meanwhile, fine roots were more numerous on June 14, leading to a higher rate of fertilizer uptake [25; 26]. This promoted *A*. *mangium* trees in the fine root monitoring technique fertilization to grow significantly better compared to those in the early rainy season and spring fertilizations (Table 1; Fig 4).

The advantage of using chemical fertilizers (e.g., NPK of 16:16:8 in the present study) is that nutrients are both soluble and immediately available to the planted trees. The effect of fertilization is usually both direct and fast [14]. However, the disadvantage is rapid loss of fertilizer by erosion, leaching, and grass uptake. Therefore, fertilization timing becomes very important. If the timing of fertilizer application is optimal, the fertilizer uptake by planted trees is rapid, which could reduce fertilizer loss. A difference of 13 days was long enough for a higher loss rate in early rainy season fertilization compared to that in fine root monitoring technique fertilization. The loss of applied fertilizers due to runoff was clearly indicated in oil palm plantation, which may reach 15% [44]. While less than 30% of N fertilizer applied was uptake by pine trees [45; 46]. Types of applied fertilizers also significantly affected the loss rates [44, 47]. The date of optimal fertilization timing for a specific species and area may change year by year, depending on climate conditions, especially rainfall. Therefore, in practical application, scanning to observe fine root growth should be carried out only in the rainy/growing season rather than throughout the whole year.

A heavy rain was recorded at the present study site on June 4–5, 2018, and this led to increased loss of fertilizer applied on June 2 through erosion and leaching. The heavy rain increased soil moisture considerably and reduced the compactness of soil after the long dry season (Fig 1B). It can be considered as a turning point that accelerated fine root growth [48; 49]. Therefore, numerous fine roots were observed on scanned images on June 10 and June 14, 5–10 days after the heavy rain. This suggests that forest plantation growers could apply fertilizer between one to two weeks after the first heavy rain of the rainy season if they were unable to use the fine root monitoring technique for observing fine root growth. However, the fine root monitoring technique is quite simple and economic, as compared with the cost of fertilizer loss from a large plantation area. O. Adu et al. [50] and Dannoura et al. [34] fixed an A4-scanner in a transparent plastic box and buried it to the soil for monitoring fine root growth. Despite being located in a temperate area with low precipitation, the scanner was damaged in less than a year due to water leaching into the scanner box Tran et al. [27]. In this study, empty transparent plastic boxes (Fig 2B) were buried in soil, covered by lids and a layer of soil to ensure darkness inside the box for fine root growth, and scanners were placed in the boxes only on dates scheduled for scanning (Fig 1C). This eliminated the potential impact of water leaching to the scanners, reducing both the cost and difficulty of the fine root monitoring technique application.

Increments of both height and DBH were lowest during December 2017–March 2018. This could be explained by seasonal dependence as it was winter at the study site, with soil temperatures of 14°C (Fig 1A). In spring, the growth increased, leading to increased increments of DBH until December 2018 and of height until September 2018. However, a sharp decrease in height growth during June–December in the spring fertilization and September–December in the fine root monitoring technique and the early rainy season fertilizations could suggest the importance of fertilization for height growth of *A*. *mangium*, as stem height had the largest increment immediately after fertilization (Fig 5C and 5D). Several studies have reported an increase in growing efficiency and higher enzymatic activities following fertilization [51; 52]. Fast-growing broad-leaved tree species like the acacia used in this study are more sensitive to increased availability of NPK [53]. Therefore, in this study, higher doses of fertilizer supported better growth (Table 1) and AGB (Fig 4).

AGB surpluses among the three fertilization timings with application of 200 g NPK/tree were much higher than those with 100 g NPK/tree (Fig 4). However, in terms of economics, a cost-benefit analysis should be conducted to determine a practical dose of fertilizer [54]. Otherwise, the AGB surplus may not compensate for the cost involved in doubling the dose of fertilizer applied.

## Conclusion

Applied fertilizer can be absorbed by planted trees and other vegetation. Fertilizing when planted trees can absorb fertilizer more efficiently could improve productivity. Therefore, fertilization timing is important in forest plantation. Using the fine root monitoring technique to observe fine root growth and identify fertilization timing with the maximum growth of fine roots resulted in significant increases in both growth and AGB of the acacia plantation when compared to the results obtained through traditional fertilizations conducted either in the early rainy season or in spring.

The experiment was conducted in an *Acacia mangium* plantation in northeast Vietnam by three different fertilization timings as in the spring (March 3, 2018), in the early rainy season (June 2, 2018), and at a time based on observation of fine root growth by a fine root monitoring technique (June 15, 2018). The results indicated that DBH, stem height, crown diameter, and AGB were highest in fertilization timing based on the fine root monitoring technique, reduced to the early rainy season fertilization and spring fertilization. Therefore, fine root monitoring technique could be used to identify optimal fertilization timing, when fine roots achieve maximum growth.
